# Supplementary material for: Metallothionein I/II Expression and Metal Ion Levels in Correlation with Amyloid Beta Deposits in the Aged Feline Brain
Source: Brain Sci. 2023 Jul 22;13(7):1115. doi: 10.3390/brainsci13071115 (PMC10377600; doi:10.3390/brainsci13071115)
Supplement: Supplementary file 1 [file brainsci-13-01115-s001.zip › brainsci-2493715-supplementary.pdf]

Supplementary materials

Table S1. Histopathological scoring of age-related brain lesions in mature, senior, and geriatric cats.

Case No	Neuronophagia	Satelitosis	Chromatolysis	Neuron lipofuscin deposits	Neuronal vacuolation	Neuronal necrosis and loss	Microglial lipofuscin deposits	Perivascular microglia	Spheroids	Lafora like bodies	H-E positive bodies
**4**	1	1	1	2	0	2	1	1	1	1	0
**5**	2	3	2	1	1	2	1	1	0	1	0
**6**	1	2	2	2	1	1	2	1	2	2	1
**7**	1	1	1	1	1	1	1	1	0	1	0
**8**	1	1	1	1	0	1	1	1	0	1	0
**9**	1	1	1	2	0	1	2	1	0	1	1
**10**	1	1	1	2	0	1	1	1	0	2	0
**11**	1	1	1	2	0	1	1	1	1	1	1
**12**	1	2	2	1	1	1	1	1	0	2	2
**13**	1	1	2	2	0	1	1	1	0	1	0
**14**	1	1	1	2	0	1	1	1	0	1	0
**15**	1	1	1	1	0	1	1	1	0	0	1
**16**	1	1	1	3	0	2	1	1	0	1	2
**17**	1	2	2	2	1	2	2	1	0	1	0
**18**	1	1	2	1	0	2	2	1	0	2	0
**19**	1	2	2	2	0	1	2	1	0	1	0
**20**	2	2	2	3	0	2	2	2	1	3	0
**21**	2	2	2	3	0	2	3	2	0	2	0
**22**	1	1	2	2	0	1	2	1	0	1	0
**23**	3	3	3	3	2	3	3	3	0	2	2
**24**	3	3	3	3	2	3	3	3	2	2	1
**25**	2	2	2	3	1	2	2	2	0	3	1
**26**	3	3	3	3	1	2	3	2	0	2	1
**27**	3	3	3	3	0	2	3	2	0	3	2
**28**	2	2	2	3	0	2	3	1	0	2	1
**29**	2	2	2	2	0	2	2	1	1	1	2
**30**	2	2	2	3	0	2	3	1	2	2	1

Case No	Neuraxonal degeneration	WM & GM vacuolation	GM&WM vascular hyalinosis	GM&WM vascular fibrosis	Leptomeningeal fibrosis	LV fibrosis	LV hyalinosis	LV calcification	CPV & CP epithelial fibrosis	CPV hyalinosis	Hemorrhages
**4**	1	2	1	1	1	2	0	0	2	0	0
**5**	2	3	1	1	2	2	0	0	1	0	0
**6**	2	1	0	0	1	2	0	0	1	0	0
**7**	1	1	0	0	0	0	0	0	0	0	0
**8**	1	2	0	0	0	0	0	0	0	0	0
**9**	1	1	0	1	1	1	0	0	1	0	1
**10**	1	1	0	0	0	0	0	0	0	0	0
**11**	1	1	0	0	0	0	0	0	1	0	1
**12**	1	1	0	0	1	1	0	1	1	0	1
**13**	1	2	0	0	1	1	0	1	1	0	0
**14**	1	1	0	1	1	1	0	1	1	0	0
**15**	1	1	0	0	1	1	0	0	1	0	0
**16**	1	2	1	1	2	2	0	0	2	0	0
**17**	1	1	1	1	1	2	0	0	1	0	0
**18**	2	1	0	1	1	2	0	0	2	1	0
**19**	1	1	0	0	2	2	0	1	3	0	0
**20**	2	2	0	1	2	2	0	0	2	0	0
**21**	2	2	1	1	2	2	1	1	3	1	0
**22**	1	1	1	0	1	1	0	0	1	0	0
**23**	3	3	2	1	3	3	2	0	3	1	0
**24**	3	3	2	1	3	3	2	1	3	0	1
**25**	3	3	1	0	1	2	1	1	3	0	0
**26**	3	2	1	0	3	3	0	0	2	0	1
**27**	3	2	1	1	3	2	0	0	3	0	0
**28**	3	2	0	1	2	2	0	0	2	0	0
**29**	2	2	1	1	2	2	0	0	2	1	0
**30**	2	1	0	1	2	2	0	0	3	1	1

Table S2. Iron deposition scoring in the brain of the aged cats.

	Temporal lobe			*Hippocampus*		*Thalamus*		*Striatum*		*Frontal lobe*			*Cerebellum*		
Case No.	**GM&WM cells**	**GM&WM IPs**	**WM MF**	**Hipp cells**	**Hipp IPs**	**Thal cells**	**Thal IPs**	**Str cells**	**Str IPs**	**GM&WM cells**	**GM&WM IPs**	**WM MF**	**GM&WM&DN cells**	**GM&WM&DN IPs**	**WM MF**
**1^{α}**	1	2	0	3	0	1	1	0	1	1	1	0	1	1	0
**2^{α}**	2	1	0	1	1	1	1	0	1	0	1	0	1	2	1
**3^{α}**	1	1	0	1	0	1	1	0	0	1	1	0	1	0	0
**4**	1	2	0	1	1	1	1	0	1	1	2	0	2	3	1
**5**	2	1	0	3	0	1	1	2	1	2	0	0	2	1	2
**6**	3	2	3	3	1	1	2	1	1	3	3	2	3	2	1
**7**	1	1	0	1	1	1	1	1	1	1	1	1	1	2	1
**8**	0	1	0	1	0	0	1	0	1	1	1	1	1	1	0
**9**	2	3	1	3	2	1	2	1	1	1	0	0	2	3	2
**10**	0	1	0	0	1	0	1	0	1	0	1	0	1	1	0
**11**	1	1	1	1	1	0	1	1	1	1	1	1	1	2	2
**12**	0	2	0	1	1	1	1	0	0	1	1	1	1	1	1
**13**	1	2	1	1	1	1	1	0	0	1	1	0	1	1	0
**14**	1	2	1	1	1	1	2	1	1	1	2	2	1	1	1
**15**	0	1	1	1	1	0	2	0	1	0	1	1	1	1	1
**16**	1	1	0	1	1	1	1	0	1	1	2	0	2	3	1
**17**	1	2	2	1	1	1	2	0	1	0	1	0	0	2	0
**18**	1	2	1	1	1	1	1	1	1	1	1	0	1	3	1
**19**	1	3	0	1	2	1	3	1	1	1	2	2	2	2	1
**20**	1	1	0	2	1	1	1	1	0	2	2	1	2	3	2
**21**	3	3	3	3	2	1	3	1	1	1	1	1	2	2	3
**22**	1	1	3	1	0	0	1	0	0	1	1	1	1	1	1
**23**	2	2	1	1	1	3	1	1	0	2	2	2	1	1	3
**24**	2	2	1	3	1	1	2	1	2	2	2	0	1	3	3
**25**	2	3	2	2	2	1	3	1	1	1	3	2	3	2	3
**26**	3	3	3	2	1	1	3	1	1	1	2	3	3	1	3
**27**	3	3	3	3	3	1	3	2	1	2	2	3	2	3	3
**28**	1	2	1	1	1	1	3	0	1	1	1	2	1	3	2
**29**	1	1	0	2	1	1	2	0	1	1	3	1	2	1	3
**30**	0	3	0	1	1	2	3	0	1	1	3	1	2	2	3

* **ves:** vessels**; Hip:** hippocampus**, Thal:** thalamus**; Str:** striatum**; MF:** myealinated fibers; **DN**: dentate nucleus

Table S3. Scoring of MT-I/II immunolabelling in cats of different ages

	Temporal lobe				*Hippocampus*			*Thalamus*		*Striatum*			*Frontal lobe*					*Choroid plexus*		*Ependyma*
Case No.	**GM**	**WM**	**LV**	**GM&WM ves**	**HIip GM**	**HIip WM**	**Hip_ves**	**Thal**	**Thal ves**	**Str**	**Str_ves**	**IC**	**GM**	**WM**	**LC**	**LV**	**GM&WM ves**	**CP**	**CP ves**	**Ependymal cells**
1[a]	0	0	1	1	1	1	0	1	1	0	0	1	1	1	0	1	0	0	1	1
2[a]	1	1	1	1	0	0	0	0	0	0	0	1	1	1	0	1	1	0	1	0
3[a]	1	1	1	1	2	2	0	2	1	1	1	2	2	2	0	1	0	1	1	1
4	1	1	1	1	1	1	0	1	0	1	0	1	1	2	0	1	0	1	1	1
5	1	1	1	1	1	1	1	1	1	0	1	1	2	3	1	1	0	1	1	1
6	1	1	1	0	2	2	0	1	0	1	0	1	1	1	1	1	0	0	1	1
7	1	1	1	1	2	2	1	1	1	1	1	1	1	1	0	1	1	1	1	1
8	1	1	1	1	2	2	0	2	0	1	0	2	1	1	0	1	0	0	1	1
9	1	1	0	0	2	2	0	1	0	1	0	1	2	1	0	1	0	0	0	0
10	1	1	0	0	2	2	0	1	0	1	0	1	2	2	0	1	1	0	0	0
11	1	1	1	1	2	2	0	1	0	1	0	1	2	1	0	1	0	1	1	1
12	1	1	1	1	2	2	0	1	0	1	0	1	1	2	0	0	0	0	1	1
13	1	1	1	1	2	2	0	1	0	1	0	2	1	2	0	1	0	0	0	1
14	1	1	1	1	2	2	0	2	0	1	0	2	2	2	0	1	0	0	0	1
15	1	2	1	1	2	2	0	2	0	1	0	2	1	1	0	1	0	0	0	1
16	1	2	1	1	1	1	0	1	1	1	1	1	1	2	1	1	0	1	1	1
17	2	2	1	1	2	2	1	2	1	1	1	1	2	2	0	1	0	1	1	1
18	2	2	1	1	2	2	1	2	1	1	1	2	1	2	0	1	0	1	1	1
19	1	2	1	1	2	2	0	1	1	1	1	2	2	2	0	1	1	1	1	1
20	1	2	1	1	3	3	1	2	1	2	1	2	3	3	0	1	1	1	1	1
21	1	3	1	1	2	2	0	2	0	1	0	2	3	3	1	1	0	1	1	1
22	1	1	1	1	2	2	0	1	0	1	0	2	1	2	0	1	1	1	1	1
23	3	3	1	1	3	3	1	3	1	2	1	2	3	3	1	1	0	1	1	1
24	3	3	1	1	3	2	1	3	0	2	0	2	3	3	1	1	0	1	1	1
25	2	2	1	1	2	2	1	2	1	1	1	2	2	2	_	1	1	1	1	1
26	3	2	1	1	3	3	0	3	1	2	1	2	3	3	1	1	0	1	1	1
27	2	3	1	0	2	2	0	2	0	1	0	2	3	3	1	1	1	1	1	1
28	2	3	1	1	3	3	1	3	1	2	1	2	2	2	1	1	1	1	1	1
29	1	3	1	1	3	2	1	3	1	2	1	2	2	3	1	1	0	1	1	1
30	2	3	1	1	3	2	1	3	1	2	1	2	2	3	1	1	1	1	1	1

* **ves:** vessels**; Hip:** hippocampus, **Thal**: thalamus**; Str**: striatum**; IC:** internal capsule.

Table S4. Grading of GFAP immunolabeling

	Temporal lobe			*Hippocampus*			*Frontal lobe*			*Cerebellum*		
**Case No**	**GM**	**WM**	**GL**	**Hip GM**	**Hip WM**	**Hip GL**	**GM**	**WM**	**GL**	**GM**	**WM**	**GL**
**4**	2	2	2	2	2	2	1	1	2	2	2	1
**5**	3	2	1	3	2	1	2	3	2	2	2	2
**6**	2	2	2	2	2	1	2	2	2	2	2	2
**7**	2	2	1	2	2	2	1	2	2	2	2	1
**8**	2	2	1	2	2	1	1	2	1	2	2	1
**9**	2	2	1	1	2	1	1	2	1	2	2	1
**10**	2	2	2	2	2	1	2	2	1	2	2	1
**11**	2	2	2	2	2	1	1	2	2	2	2	1
**12**	2	2	1	2	2	2	1	2	1	3	2	2
**13**	1	1	1	1	1	2	1	2	1	1	1	2
**14**	1	2	2	2	2	1	2	1	2	1	2	1
**15**	2	2	1	2	1	1	2	2	1	2	2	1
**16**	3	3	2	1	3	2	2	2	2	3	2	2
**17**	2	3	2	3	2	2	2	2	2	2	2	2
**18**	2	3	2	2	2	2	2	3	2	2	2	2
**19**	2	2	2	2	2	2	2	2	1	2	2	2
**20**	3	2	2	3	2	2	2	2	1	3	2	2
**21**	3	3	3	3	2	2	3	3	2	3	2	3
**22**	3	3	2	2	2	2	2	2	2	2	3	2
**23**	3	3	3	3	3	3	3	3	2	3	3	3
**24**	3	3	3	3	3	3	3	3	3	3	3	3
**25**	3	3	2	3	2	2	2	2	3	3	3	2
**26**	3	3	3	3	3	3	3	2	2	3	3	3
**27**	3	3	3	3	3	2	3	3	2	3	3	3
**28**	3	3	2	3	2	2	2	3	2	2	3	2
**29**	2	3	2	3	2	2	2	3	3	3	2	2
**30**	3	3	2	2	3	2	3	3	3	3	3	3

* **GL**: glia limitans; **Hip:** hippocampus

Table S5. Scoring of Aβ immunolabelling in the brain of cats of different ages

	Temporal lobe				**Hippocampus**						**CP**		**Frontal lobe**				**Cerebellum**		
**Case No**	**Leptomeningeal CAA**	**GM+WM CAA**	**GM neurons**	**GM&WM SPs**	**Hip neurons**	**Hip SPs**	**Thal neurons**	**Thal SPs**	**Str neurons**	**Str SPs**	**CPV**	**CP epithelial cells**	**Leptomeningeal CAA**	**GM&WM CAA**	**GM neurons**	**GM+WM SPs**	**Leptomeningeal CAA**	**GM+WM CAA**	**GM&DN neurons**
	***p<0,001**	***p<0,001**	***p=0.095#**	***p<0,001**	***p<0,002**	**Non-signifant**	***p<0,029**	***p=**0.092 #	**Non-signifant**	*p= 0.047	***p<0,001**	***p<0,001**	***p<0,001**	***p<0,002**	*p=0.021	***p<0,001**	***p<0,002**	***p<0,001**	***p<0,001**
**4**	1	1	0	3	1	1	1	0	1	0	2	3	3	3	1	1	0	0	0
**5**	1	1	0	3	1	0	1	0	1	0	3	2	3	1	2	1	0	0	0
**6**	1	3	1	3	1	0	1	0	1	0	3	3	3	1	2	1	0	0	0
**7**	2	3	1	0	1	0	1	0	1	0	1	1	2	2	1	0	3	1	1
**8**	1	1	1	0	1	0	1	0	0	0	1	1	3	1	1	0	3	3	1
**9**	3	3	0	0	1	0	0	0	0	0	1	1	2	1	1	0	3	2	1
**10**	1	2	1	0	1	0	1	0	1	0	2	2	2	3	1	0	1	1	1
**11**	1	1	1	0	1	0	1	0	1	0	1	2	2	1	1	0	1	1	1
**12**	2	1	1	0	1	0	1	0	1	0	2	3	2	1	1	0	1	1	1
**13**	2	2	1	1	1	1	1	0	1	0	2	2	3	1	1	1	3	3	1
**14**	2	3	1	1	2	0	1	0	1	0	1	2	3	2	1	1	1	3	1
**15**	2	2	0	1	0	0	0	0	0	0	1	1	1	0	0	0	2	1	1
**16**	3	1	0	2	0	0	0	0	0	0	2	2	3	2	1	0	2	2	1
**17**	2	3	1	3	2	0	1	0	1	0	2	2	3	3	1	1	1	1	1
**18**	2	1	1	3	2	0	1	3	2	1	2	3	2	2	2	3	0	0	0
**19**	2	1	1	2	2	1	1	0	0	0	2	3	2	3	1	1	1	1	1
**20**	2	2	0	2	2	0	1	0	1	1	2	2	3	2	1	2	2	2	1
**21**	1	1	0	3	1	1	1	3	1	3	3	3	3	1	1	3	3	3	1
**22**	2	1	1	1	1	0	1	0	1	0	1	2	3	1	1	0	1	1	1
**23**	3	3	1	3	2	1	1	2	1	2	3	3	3	3	2	3	3	2	1
**24**	2	1	1	3	2	1	1	2	1	1	2	3	3	3	1	3	2	2	1
**25**	1	1	0	3	0	0	0	3	0	1	2	2	3	1	1	3	1	2	1
**26**	1	1	0	3	2	0	1	3	1	0	3	3	3	2	1	3	3	2	1
**27**	1	1	0	3	1	3	1	1	1	2	1	3	2	1	0	3	3	3	2
**28**	1	1	0	3	1	1	0	0	0	1	1	2	3	2	1	3	2	2	1
**29**	1	1	0	3	0	2	0	0	0	3	2	2	2	2	0	3	3	3	2
**30**	2	1	0	3	2	3	1	0	1	0	3	3	3	2	1	3	2	2	1

* **ves:** vessels; **Hip:** hippocampus, **Thal:** thalamus; **Str:** striatum.

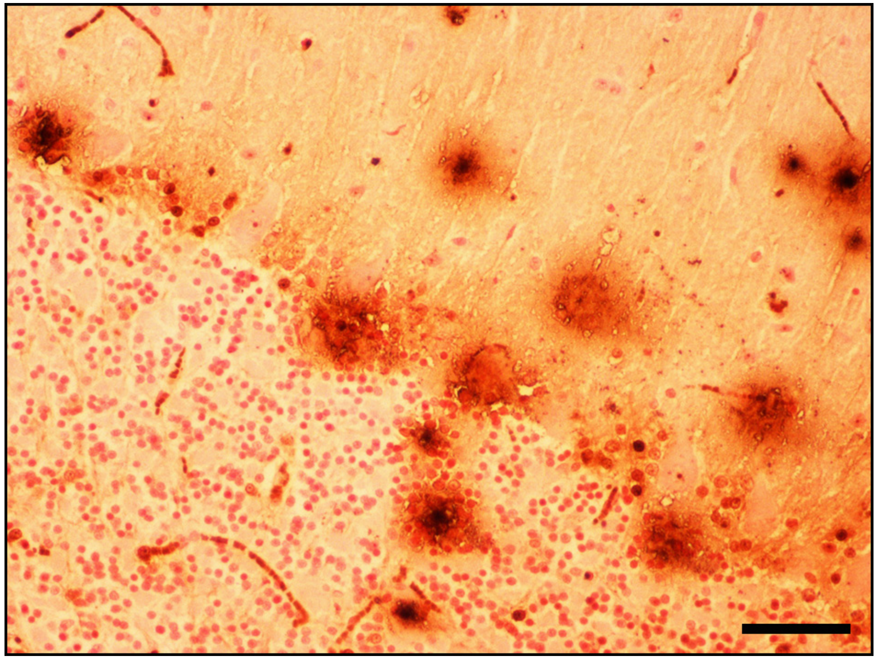

Figure S1. Condensed plaques characterized by spherical accumulation of iron with dense core and diffuse, poorly delimited IPs. Neurons and glial cells are also stained. Perl's/DAB. Bar=50μm
